# The relationship between depressive mood and non-suicidal self-injury among secondary vocational school students: the moderating role of borderline personality disorder tendencies

**DOI:** 10.3389/fpsyt.2023.1187800

**Published:** 2023-10-05

**Authors:** Zhaoyuan Lu, Mo Chen, Shu Yan, Weixi Deng, Taimin Wu, Lianzhong Liu, Yang Zhou

**Affiliations:** ^1^School of Medicine, Jianghan University, Wuhan, China; ^2^Department of Psychiatry, Wuhan Mental Health Center, Wuhan, China; ^3^Affiliated Wuhan Mental Health Center, Jianghan University, Wuhan, China; ^4^Tongji Medical College of Huazhong University of Science and Technology, Wuhan, China; ^5^Institute of Education, China University of Geosciences (Wuhan), Wuhan, China; ^6^Affiliated Wuhan Mental Health Center, Tongji Medical College of Huazhong University of Science and Technology, Wuhan, China

**Keywords:** non-suicidal self-injury (NSSI), depression, personality disorder, adolescent, personality tendencies, moderating effect

## Abstract

**Background:**

Non-suicidal self-injury (NSSI) has become an important public health issue of global concern, often occurring in adolescents, and depressive mood is closely related to NSSI. In addition, NSSI is considered a symptom of borderline personality disorder. It has been found that adolescents in secondary vocational schools are more vulnerable to behavior and emotional disorders than those in general high schools. This study investigated the risk factors associated with NSSI affecting secondary vocational school students and analyzed the role of borderline personality disorder tendencies in promoting the occurrence of NSSI among students with depressive moods.

**Methods:**

A total of 1,848 Chinese secondary vocational students completed a self-report questionnaire. The homemade NSSI behavior questionnaire, Patient Health Questionnaire-9 and Personality Diagnostic Questionnaire-4 were used in this survey. Binary logistic regression and PROCESS software analysis were used to explore the influencing factors associated with NSSI and to test for moderating effects.

**Results:**

Female (OR = 3.412, 95% CI 2.301–5.060), drinking history (OR = 2.007, 95% CI 1.383–2.911), history of suicidal death exposure (OR = 3.161, 95% CI 1.999–4.999), depressive mood (OR = 2.436, 95% CI 1.668–3.558) and borderline personality disorder tendencies (OR = 2.558, 95% CI = 1.764–3.711) were independent risk factors for NSSI. Borderline personality disorder tendencies (B = 0.047, *p* = 0.000) moderated the relationship between depressive mood and NSSI. The stronger the borderline personality tendencies, the more NSSI behavior occurred when they were depressive.

**Conclusions:**

Borderline personality disorder tendencies in secondary vocational school adolescents significantly enhance the association of depressive mood with NSSI. There is a moderating role for borderline personality disorder tendencies in depressive mood and NSSI.

## Introduction

Non-suicidal self-injury (NSSI) refers to intentional and direct injury to the individual’s body without the intention of suicide; it is not socially acceptable and often occurs in adolescents (1). In a study of nonclinical adolescents from 2010 to 2021, common types of NSSI were found to include banging/hitting, pinching and pulling of hair, with less drug-taking behavior (2). The results of meta-analysis of the prevalence of NSSI by Sarah V Swannell and other researchers showed that the prevalence of NSSI among adolescents in the community was about 17.2% (3). In addition, NSSI is a common feature of suicide attempts and can significantly predict suicidal behavior (4).

Previous research exploring NSSI has focused on hospitals, community colleges, or high schools, with less attention paid to secondary vocational school students (5). According to the Key Results of China’s National Education Statistics in 2021 (6), in 2021, more than 13 million students enrolled in secondary vocational schools, which is a large student population. In China, after graduating from junior high school, students with low scores on the secondary school entrance exam will attend a secondary vocational school. Students will learn vocational skills while in school and will most likely to work directly after graduation rather than pursue further education. Compared with general high schools, secondary vocational students may show more emotional and behavioral problems due to differences in parenting style, academic performance, and sociocultural background (7, 8). Students in secondary vocational schools reported higher rates of smoking, drinking, fighting, running away from home, prolonged Internet use, Internet addiction, and sexual activity than students in regular high schools (9, 10).Therefore, they need more attention.

Psychological factors, especially personality traits, play a very important role in the onset, development and prognosis of mental disorders (11). NSSI has been found to be very common in patients with personality disorders, especially borderline personality disorder (12). In addition, NSSI seems to predict the development of BPD. A significant longitudinal association between NSSI and later diagnosis of BPD and critical symptoms was found in adolescents followed up between the ages of 15–17 years (13). Borderline personality disorder (BPD) is a psychiatric-psychological disorder. In the Diagnostic and Statistical Manual of Mental Disorders (DSM-5) (14), it is classified as a category B personality disorder and is centered on emotional specificity. Patients with BPD can exhibit instability with respect to cognition, emotion, behavior, etc. In addition, more than 75% of patients with BPD are self-injurious or suicidal (15). It has been shown that BPD symptoms is associated with NSSI in nonclinical samples and that the higher the propensity for BPD, the higher the probability of self-injury (16). Recent findings have shown that patients with depression and self-injury exhibit more borderline symptoms (17).

Studies have found that people with depression are the largest population with NSSI, and adolescents are at high risk for NSSI yet the most vulnerable. The prevalence of NSSI in Chinese adolescents with depression is reported to be 42.50% (18). Depressive disorder is a major risk factor for NSSI, and both conditions can predict each other (19–21). In a cross-sectional study of Korean high school students (22), the results showed that high depression levels were significantly associated with NSSI. In addition, epidemiologic studies have found that adolescents with borderline personality traits report depression approximately 18–25 times more often than adolescents without borderline personality traits. Borderline personality traits show a high degree of co-morbidity with depression (23). Depression is highly comorbid with BPD, with over 85% of BPD patients presenting with symptoms that overlap with those of MDD patients (24, 25). Depressive symptoms are common in patients with BPD, and antidepressant medications are helpful in the treatment of BPD (26, 27). In addition, depression and borderline personality traits have common features. For example, both have symptoms associated with rumination. Rumination was not only positively correlated with depression and anxiety, but also significantly correlated with features of instability and inconsistent self-consciousness in borderline personality disorder (28).

Depression or BPD may be co-morbid with NSSI (10). The reason for this may be that patients with both depressive disorders and BPD may have negative emotions and poor interpersonal relationships, and NSSI moderates these negative emotions and helps them gain more interpersonal attention (26). A cross-sectional study found that BPD traits have an important role in depression and NSSI. In the study analysis, after controlling for BPD, the total effect of depression on the interpersonal motivation-related NSSI was found to be greater than the effect on the mood-related NSSI, in other words,BPD features may be more prominent in depressed individuals who engage in interpersonal NSSI (26).

There is growing evidence that depressive mood, BPD, and NSSI are related. Despite these findings, to the best of our knowledge, no study has explored the specific role that BPD tendency plays in the relationship between depressed mood and NSSI. The relationship between these three variables, NSSI and depressed mood and BPD tendency has also never been explored among Chinese secondary vocational school students. There are fewer relevant studies quantifying the role of BPD in depression and NSSI, and it is unclear whether there is a moderating role of BPD tendencies in the relationship between depressive mood and NSSI in secondary vocational school students.

Thus, this study aims to explore the risk factors associated with NSSI in secondary vocational school students, and investigate the role of BPD tendencies in depressive mood and NSSI risk. We hope to find the relevant factors influencing NSSI in secondary vocational school students.

## Method

### Participants

The participants of this survey were recruited from a secondary vocational school in Wuhan. The study adopts cluster sampling. We sent a total of 2,160 questionnaires, all of which were returned, and 312 questionnaires with incomplete information and unreliable answers were excluded, resulting in 1848 valid questionnaires. The validity rate was 85.5%. The age range of students was 16–18 years old, among which 802 (43.4%) were male and 1,046 (56.6%) were female. There were 844 (45.7%) high school freshmen, 756 (40.9%) high school sophomores, and 248 (13.4%) high school juniors.

### Procedures

Participants were recruited through a secondary school in Wuhan. Before the investigation, we first contacted the school director and teachers. Parents had to complete consent. After obtaining consent, we distributed the questionnaire to children whose parents agreed to participate in the survey.

Before formally conducting the questionnaire, a presurvey was conducted to refine the shortcomings of the questionnaire. The survey used a self-administered questionnaire. The test was conducted in a class unit. The investigator will read out the instructions and explain the purpose and significance of the survey to the students. After the unified distribution of the questionnaire, the class teacher will assist in supervising the test subjects and require them to fill it out independently on the spot. The time should not exceed 25 min. If there is any doubt about the content of the questionnaire, either the investigator or the class teacher can provide guidance. Questionnaires with obvious logical errors or omission rates >15% were excluded.

### Measures

#### Sociodemographic profile

A self-administered questionnaire was used to collect personal information from students. This included gender, grade level, whether the student was an only child, parents’ education level, family financial situation, parents’ marital status, whether they lived with their parents, whether they smoked or drank alcohol, whether anyone in their immediate family had been diagnosed with a mental/psychiatric illness, whether any of their relatives/friends or other acquaintances had passed away due to suicide, etc.

#### Measurements of NSSI

The assessment of NSSI behavior in this study was conducted with one item, “In the past 12 months, have you intentionally injured yourself but not attempted to commit suicide?.” The participants were asked to answer yes or no according to their actual experience (yes = 1, no = 0). If students choose yes, they need to answer the question “About how many times have you hurt yourself?” (1–2 times = 1, 3–5 times = 2, 6–8 times = 3, more than 8 times = 4).

#### Patient health questionnaire-9 (PHQ-9)

Depressive mood was assessed using the PHQ-9. It contains nine items: (1) anhedonia; (2) depressed mood; (3) trouble sleeping; (4) feeling tired; (5) change in appetite; (6) guilt, self-blame, or worthlessness; (7) trouble concentrating; (8) feeling slowed down or restless; and (9) thoughts of being better off dead or hurting oneself (29). Each item is rated on a 4-point scale from 0 to 3 (0 = never, 1 = several days, 2 = more than half the time, and 3 = nearly every day) during the 2 weeks prior to and including the day of survey completion. The total score ranges from 0 to 27, and a score of 10 or greater represents depressive symptoms of at least moderate severity and is the most commonly used cutoff point when screening for major depressive disorder. The Chinese version of the PHQ-9 used in this study has reported great reliability and validity; the Cronbach’s alpha value was 0.85, and the 4-week test–retest reliability was 0.88. The reliability and validity have been confirmed in the Chinese adolescent population (30).

#### Personality diagnostic questionnaire-4 (PDQ-4)

The PDQ is a self-report questionnaire based on the DSM-III screening for personality disorders developed by Dr. Hyler in the U.S. and revised in 1988 (PDQ-R).The PDQ-R has a retest agreement rate of 77.78% (31). Dr. YANG J revised the PDQ-4 into a Chinese version (32). It consists of 107 entries. The Chinese version of the BPD subscale of the PDQ-4 was used in this study. The BPD subscale has 11 entries (yes = 1, no = 0). It has 2 entries that use topics outside of parentheses as marking items, 100 (6) and 101 (19). Question 106 contains 6 sub-topics, with sub-topics greater than or equal to 2 answered “yes” being scored as 1, otherwise 0. The BPD subscale encompasses multiple dimensions such as emotional instability, relationship instability, impulsivity, dissociative disorders, self-injury and self-harm. Yueqin H (33) pilot tested it in a population sample of Chinese secondary vocational school students, and the results showed that the PDQ-4 had good validity and reliability. Later, Yang Y et al. tested the PDQ-4 and showed that the PDQ-4 has high sensitivity and low specificity, with a retest reliability of 0.50–0.80. The BPD subscale retest reliability coefficient was 0.79, and can be used to screen for personality disorders (34). The entries add up to a total score between 0 and 9. Positive BPD tendencies was classified as 5 points.

### Statistical analysis

The study used SPSS 26.0 software for statistical analysis of the data. Count data were described using composition ratios or rates. The *X*^2^ test or Fisher’s exact probability method was selected for comparisons between groups. Binary logistic regression was used for the analysis of NSSI influencing factors. Moderating effects were performed with the PROCESS software (Model 1) installed on SPSS to investigate the moderating effect of BPD tendencies (moderating variable) between depressed mood (independent variable) and NSSI (dependent variable) (35). The plotting tool was also used to characterize the simple slopes of the study results (36). In the analysis, a difference was considered statistically significant at *p* < 0.05.

## Results

### Preliminary analyses

In this survey, 1848 secondary vocational school students were investigated. Among them, 186 had NSSI, and the prevalence was 10.06%. The detection rate of NSSI was 5.4% for males and 13.7% for females, with statistically significant differences (*p* < 0.05). There were statistically significant differences in grade, family economic status, single-parent family, whether they lived with their parents during childhood or now, smoking history, drinking history, whether their immediate family members had mental disorders, and whether their relatives and friends had passed away by suicide (*p* < 0.05). Detailed information on the differences between the NSSI and non-NSSI groups can be found in Table 1.

**Table 1:** Characteristics of participants by NSSI, *n* (%): A secondary vocational school survey in Wuhan *n* = 1848.

Variables	Total	NSSI		*X^2^*	*p* value
		Yes	No		
*Gender*					
Male	802 (43.4%)	43 (5.4%)	759 (94.6%)	34.627	0.000
Female	1,046 (56.6%)	143 (13.7%)	903 (86.3%)		
*Grade*					
Senior one	844 (45.7%)	106 (12.6%)	738 (87.4%)	10.712	0.005
Senior two	756 (40.9%)	61 (8.1%)	695 (91.9%)		
Senior three	248 (13.4%)	19 (7.7%)	229 (92.3%)		
*Only child*					
Yes	945 (51.1%)	92 (9.7%)	853 (90.3%)	0.232	0.630
No	903 (48.9%)	94 (10.4%)	809 (89.6%)		
*Father’s education level*					
Primary school or less	171 (9.3%)	18 (10.5%)	153 (89.5%)	0.888	0.641
Junior and senior high school	1,290 (68.8%)	134 (10.4%)	1,156 (89.6%)		
College or more	387 (20.9%)	34 (8.8%)	353 (91.2%)		
*Mother’s education level*					
Primary school or less	269 (14.6%)	27 (10.0%)	242 (90.0%)	0.044	0.978
Junior and senior high school	1,211 (65.5%)	123 (10.2%)	1,088 (89.8%)		
College or more	368 (19.9%)	36 (9.8%)	332 (90.2%)		
*Economic status of family*					
Very poor	41 (2.2%)	5 (12.2%)	36 (87.8%)	13.021^*^	0.010^*^
Poor	175 (9.5%)	30 (17.1%)	145 (82.9%)		
Moderate	1,266 (68.5%)	117 (9.2%)	1,149 (90.8%)		
Good	306 (16.6%)	32 (10.5%)	274 (89.5%)		
Very good	60 (3.2%)	2 (3.3%)	58 (96.7%)		
*Single-parent family*					
Yes	308 (16.7%)	42 (13.6%)	266 (86.4%)	5.208	0.022
No	1,540 (83.8%)	144 (9.4%)	1,396 (90.6%)		
*Lived with their parents during childhood*					
Yes	1,022 (%)	85 (8.3%)	937 (91.7%)	7.717	0.005
No	826 (%)	101 (12.2%)	725 (87.8%)		
*Live with their parents now*					
Yes	1,234 (66.8%)	110 (8.9%)	1,124 (91.1%)	5.489	0.019
No	613 (33.2%)	76 (12.4%)	537 (87.6%)		
*Smoking history*					
Yes	183 (9.9%)	37 (20.2%)	146 (79.8%)	23.134	0.000
No	1,665 (90.1%)	149 (8.9%)	1,516 (91.1%)		
*Drinking history*					
Yes	678 (36.7%)	105 (15.5%)	573 (84.5%)	34.777	0.000
No	1,170 (63.3%)	81 (6.9%)	1,089 (93.1%)		
*Family history of psychiatric disorders*					
Yes	177 (9.6%)	33 (18.6%)	144 (81.4%)	15.916	0.000
No	1,671 (90.4%)	153 (9.2%)	1,518 (90.8%)		
*History of suicidal death exposure*					
Yes	159 (8.6%)	46 (28.9%)	113 (71.1%)	68.404	0.000
No	1,689 (91.4%)	140 (8.3%)	1,549 (91.7%)		

^*^Using Fisher’s exact probability method.

Among 1848 secondary vocational school students, there were 443 (24.0%) without depression, 1,079 (58.4%) with mild depression, and 326 (17.6%) with moderate to severe depression. The prevalence of moderate to severe depression was higher among female students (10.6%) than male students (7.0%). The percentage of students with moderate to severe depressive mood was highest in the senior one class (50.6%), followed by senior two (39.0%) and senior three (10.4%) grades. There were 348 (18.8%) students with positive BPD tendencies, including 123 males and 225 females. Half of them were senior one students, while senior two and senior three accounted for 40.0 and 10.0%, respectively.

Totally, 186 secondary vocational school students had NSSI. A chi-square test was used to compare the difference between the incidence of NSSI among secondary school students with or without BPD tendencies. As shown in Table 2, the incidence of NSSI in secondary school students without BPD tendencies was 6.5%, which was significantly higher than that in students with a positive BPD tendencies (25.3%) (*p* < 0.001).

**Table 2:** NSSI differences under different BPD tendencies.

Variables	Total	NSSI (Yes)	NSSI (No)	*X^2^*	*p* value
BPD tendencies (Yes)	348 (18.8%)	88 (25.3%)	260 (74.7%)	109.753	0.000
BPD tendencies (No)	1,500 (81.2%)	98 (6.5%)	1,402 (93.5%)		

### Analysis of NSSI impact factors

Binary logistic regression was used to analyze the risk factors associated with influencing NSSI. At this point, we used categorical variables to assess depressed mood and BPD tendencies. The results showed that adolescents who were female (OR = 3.412, 95% CI 2.301–5.060), had drinking behaviors (OR = 2.007, 95% CI 1.383–2.911), had relatives or friends who had passed away by suicide (OR = 3.161, 95% CI 1.999–4.999), who were depressive (OR = 2.436, 95% CI 1.668–3.558) and who were prone to BPD (OR = 2.558, 95% CI = 1.764–3.711), were more likely to engage in self-injurious behavior (*p* < 0.05) (Table 3).

**Table 3:** NSSI-related influences.

Variables	β	S.E.	Wals	df	*p* value	Exp (B)	EXP (B) 95% CI	
							Lower bound	Upper bound
Female	1.227	0.201	37.249	1	0.000	3.412	2.301	5.060
Drinking history	0.697	0.190	13.471	1	0.000	2.007	1.383	2.911
History of suicidal death exposure	1.151	0.234	24.214	1	0.000	3.161	1.999	4.999
Depressive mood	0.890	0.193	21.224	1	0.000	2.436	1.668	3.558
BPD tendencies	0.939	0.190	24.518	1	0.000	2.558	1.764	3.711

### Moderating effect of BPD tendencies

To test whether BPD tendencies moderated the relationship between depression and NSSI, we used single-model moderation analyses in PROCESS. PROCESS was used for creating the hierarchical regression model. All results are standardized except for the dependent variable results. Regarding the dependent variable, we approximate the rank variable related to frequency as a continuous type variable. Besides, continuous type scores were used to assess the propensity for BPD. It is mean above 1 SD classified as high BPD tendencies. One SD below the mean is below the minimum observed in the data for BPD tendencies, so the minimum measurement on BPD tendencies is used for conditioning instead.

Findings indicated that BPD tendencies did significantly modulate the association between depressed mood and NSSI (B = 0.047, *p* = 0.000). There was a moderating role of BPD tendencies in depressive mood and NSSI (Table 4).

**Table 4:** Examination of the moderating effect of BPD tendencies on depressive mood and NSSI.

	*B*	*SE.B*	*t*	*p*	*LLCI*	*ULCI*
(Constant)	0.155	0.015	10.465	0.000	0.126	0.185
Depressive mood	0.126	0.016	7.680	0.000	0.094	0.158
BPD tendencies	0.076	0.017	4.457	0.000	0.043	0.110
Depressive mood × BPD tendencies	0.047	0.012	3.966	0.000	0.024	0.070
Model summary	*F* = 88.437, *p* = 0.000, *R*^2^ = 0.126					

B, unstandardized coefficient; SE. B, standard error; *t*, *t*-test score; *p*, *p* value; LLCI, lower-level confidence interval; ULCI, upper-level confidence interval; *F*, *F*-test.

**Figure 1:**
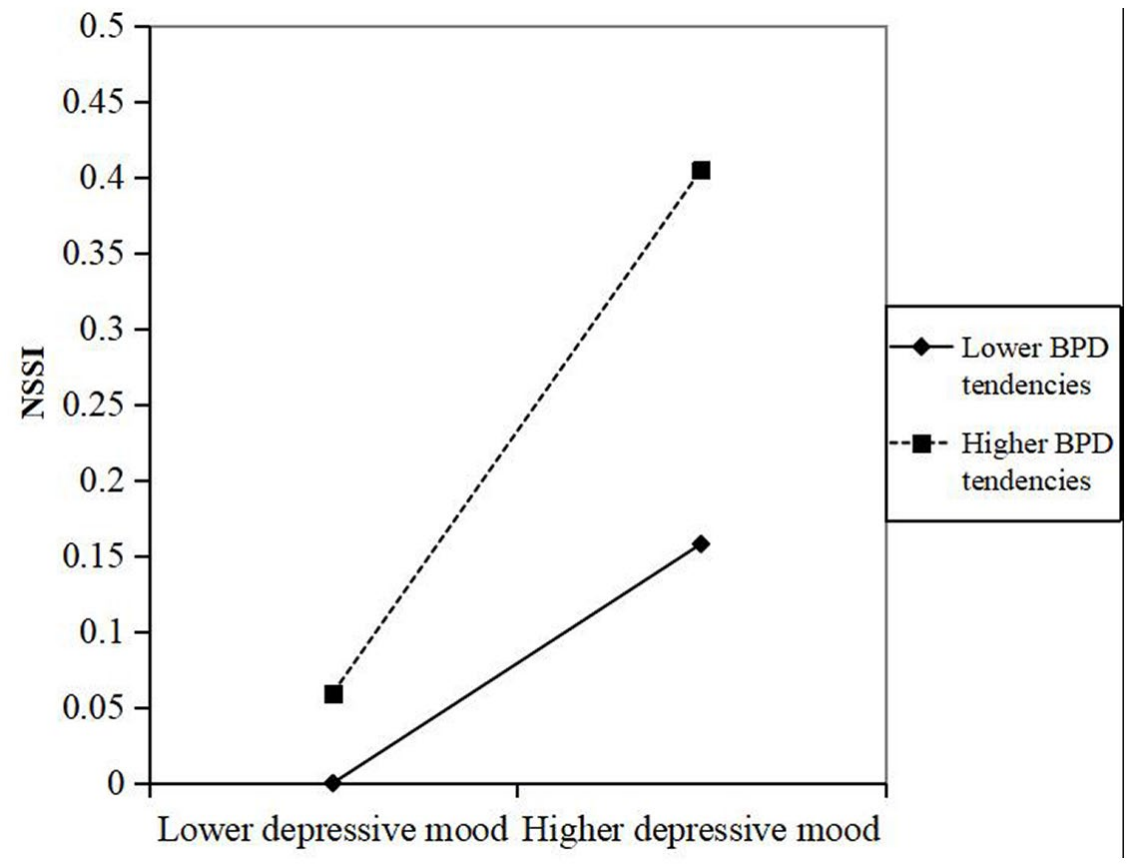
Moderating role of BPD tendencies between depressive mood and NSSI.

To more visually represent the moderating effect of BPD tendencies, the moderating effect was plotted in this study. As shown in Figure 1, overall, the results of the study showed that there was a positive correlation between depressed mood and NSSI, both among participants with a high or low tendency toward BPD. The effect of depressive mood on NSSI was attenuated in participants with low BPD tendencies (B = 0.079, *p* = 0.001) compared to participants with high BPD tendencies (B = 0.173, *p* = 0.000). To put it simply, the positive effect of depressive mood on NSSI was greater when BPD tendencies was at higher level and more likely to have NSSI, indicating that adolescents with high depressive mood and high BPD tendencies exhibited more NSSI behaviors.

## Discussion

The results of this study showed that the prevalence of NSSI was 10.06% among 1848 students in a secondary vocational school in Wuhan, which was lower than the total prevalence of NSSI among Chinese secondary vocational school students (22.37%) obtained by Lang et al. in their meta-analysis (37). It may be influenced by the sample source, year region, measurement method and criteria (3, 19, 38, 39). An anonymous questionnaire was used in this study. However, it does not exclude reports of students who have a sense of shame or other concerns about hiding NSSI behaviors when completing the questionnaire in a classroom setting (40).

This study showed that the prevalence of NSSI was higher in women than in men (13.7% > 5.4%). This is similar to the results of the study by Monto et al. (41)_,_ which concluded that female was an independent risk factor for NSSI (OR = 3.412, 95% CI 2.301–5.060), possibly because women are more emotionally sensitive, prone to negative emotions and more likely to experience more negative events (2).Whereas, Some studies have taken the opposite view, suggesting that men are more prone to NSSI behaviors because they are reluctant to ask for help and tend to externalize their internal emotions when coping with negative events (42, 43). The risk of suicide after self-injury is also much higher in men than that in women (44). Researchers have suggested that NSSI behaviors tend to occur in mid-adolescence, usually between the ages of 14 and 17 (45), and begin to decline in mid- to late-adolescence (20). In this study, the prevalence of NSSI also showed a decreasing trend from freshman to junior year. This may be related to the dramatic changes in adolescents’ physical and mental development during adolescence and maladjustment from middle school to high school (43). Secondary vocational school students with low family economic level, divorced or widowed parents, and those who did not live with their parents in childhood or at present have a higher probability of NSSI behavior. These results suggest that an affluent, warm, and loving parental home environment can help prevent the occurrence of NSSI in adolescents (46). The rates of smoking and drinking students were higher in the NSSI group than in the non-NSSI group. The occurrence of NSSI was 2.007 times (OR = 2.007, 95% CI 1.383–2.911) higher in drinking students than in nondrinking students. Smoking and alcohol consumption are associated with adverse life events (47, 48). And these events can increase the risk of self-injury (49). The current study also found that adolescents with a family history of psychiatric illness or exposure to suicide deaths (OR = 3.161, 95% CI 1.999–4.999) may be more prone to NSSI. Students may imitate the self-injurious or suicidal behavior of relatives and friends (50). In addition, because of the prevalence of self-injury and suicidal behavior around them, adolescents believe that this behavior is justified and socially acceptable (51).

Among the 1848 secondary vocational school students, the rate of students with moderate-to-severe depressive mood was 17.6% and was higher among female students than that in male students (60.1% > 39.9%), which is consistent with the findings of a previous study by Shorey et al. (52). Most likely because of biological vulnerability, female individuals respond more to depression perceptions as well as stress exposure and are more susceptible to depressive symptoms than male individuals due to other psychosocial and cultural influences. We found that the rate of students with moderate-to-severe depressive mood with positive BPD tendencies decreased with increasing grade level. However, in a study of German adolescents, Wartberg et al. (53) found that depressive symptoms were more common with increasing age, which is contrary to our findings. In addition, Videler (54) suggested that the fluctuation of BPD symptoms with age is influenced by social context and developmental factors. Students in secondary vocational schools have just gone through secondary school exams and need to adapt to a new learning environment during their first year in school. As the grade level increases, students’ study pressure will decrease. The development direction changes from further education to employment. With the increase in life experience, the improvement of self and social awareness and the strengthening of control over emotion, the depressive mood and BPD symptoms of adolescents decrease.

In this study, the rate of NSSI among students with positive BPD tendencies (25.3%) was 3.89 times higher than that of secondary vocational school students without BPD tendencies (6.5%). Logistic regression analysis showed that having a depressed mood and BPD tendencies had a significant effect on NSSI. The results of the study implies that the higher the students’ depressive mood or BPD tendencies is, the higher the likelihood that they will exhibit NSSI behaviors. Both depressed mood and BPD tendencies are associated with difficulties in emotion regulation, while NSSI helps to alleviate adverse emotions and negative cognitive states, increase positive emotions, and maintain emotional balance. When emotions remain ineffectively regulated, teenagers take more extreme approaches, such as suicide (4). Higher levels of depression or severity of BPD symptoms can predict suicide attempts (55). In addition, after the occurrence of NSSI behaviors, adolescents are more likely to experience low self-esteem, emptiness, and anger, which can increase the frequency of self-injurious behaviors, creating a vicious cycle (56).

Another aim of this study was to analyze the role of BPD tendencies in depressive mood and NSSI risk, and the findings showed that BPD tendencies can play a moderating role in depressive mood and NSSI. Specifically, we found that for secondary vocational school students with low BPD tendencies, an increase in depressive mood led to a mild increase in NSSI, whereas for students with high BPD tendencies, there was a trend of a significant increase in NSSI with an increase in depressive mood. Peters et al. (26) argued that different NSSI motivations lead to different role sizes of BPD characteristics between depression and NSSI. Emotion regulation difficulties and poor interpersonal relationships are two common motivations for NSSI. Secondary vocational school students are in adolescence. Adolescent students experience an increased incidence of internalization and externalization as they undergo dramatic psychological and physical changes, academic and employment pressures, and fluctuations in social relationships that may lead to uncontrolled emotional regulation and increased interpersonal stress (57). Moreover, BPD symptoms are mainly characterized by interpersonal, self-image and emotional instability, which can worsen emotional and interpersonal problems. Therefore, depressed students with high BPD tendencies are more likely to develop NSSI.

## Limitations

Several limitations must be discussed in this study. First, the study only sampled one secondary vocational school in Wuhan, which is just one school. The kind of research sample is single. Second, the study used only one item to measure NSSI, which may lead to inaccurate findings. In addition, the study did not assess the frequency of NSSI behaviors, patterns of self-harm, purposes of self-harm, and other related components, so future research needs to improve upon these methods. Third, the survey in this study was a cross-sectional survey, thus indicating that the results may be subject to recall bias and that causal inference cannot be made. Therefore, these results need to be verified in conjunction with prospective studies.

## Conclusion

In summary, being female, having an alcohol-drinking habit, having a history of exposure to suicide deaths, having a high depressive mood, and having a high BPD tendencies were independent risk factors for NSSI behavior among secondary vocational school students. BPD tendencies significantly strengthens the association between depressive mood and NSSI, and there is a moderating role of BPD tendencies in depressive mood and NSSI. Students in secondary vocational schools have been an easily neglected group, and various pressures from school and society may bring them a series of psycho-behavioral problems. Therefore, educators and medical professionals need to pay more attention to their psychosomatic health, identify and intervene in risk factors as early as possible, help students improve their emotional control, develop a sound personality, and reduce the occurrence of NSSI behaviors so that they can successfully complete the school-to-society transition and better adapt to society.

## Data availability statement

The raw data supporting the conclusions of this article will be made available by the authors, without undue reservation.

## Ethics statement

The studies involving humans were approved by Department of Psychiatry, Wuhan Mental Health Center. The studies were conducted in accordance with the local legislation and institutional requirements. Written informed consent for participation in this study was provided by the participants' legal guardians/next of kin. Written informed consent was obtained from the minor(s)' legal guardian/next of kin for the publication of any potentially identifiable images or data included in this article.

## Author contributions

ZL performed the data analysis and wrote the first draft of the manuscript. MC and SY conceived the study and guided the data analysis. WD and TW contribute to the data analysis. LL and YZ supervise and direct all steps of the study. All authors contributed to the article and approved the submitted version.
